# Locational Determinants of Emissions from Pollution-Intensive Firms in Urban Areas

**DOI:** 10.1371/journal.pone.0125348

**Published:** 2015-04-30

**Authors:** Min Zhou, Shukui Tan, Mingjing Guo, Lu Zhang

**Affiliations:** 1 Non-traditional Security Center of Huazhong University of Science and Technology, Wuhan, China; 2 College of Public Administration, Huazhong University of Science and Technology, Wuhan, China; 3 School of Economics and Management, China University of Geosciences, Wuhan, China; NERC Centre for Ecology & Hydrology, UNITED KINGDOM

## Abstract

Industrial pollution has remained as one of the most daunting challenges for many regions around the world. Characterizing the determinants of industrial pollution should provide important management implications. Unfortunately, due to the absence of high-quality data, rather few studies have systematically examined the locational determinants using a geographical approach. This paper aimed to fill the gap by accessing the pollution source census dataset, which recorded the quantity of discharged wastes (waste water and solid waste) from 717 pollution-intensive firms within Huzhou City, China. Spatial exploratory analysis was applied to analyze the spatial dependency and local clusters of waste emissions. Results demonstrated that waste emissions presented significantly positive autocorrelation in space. The high-high hotspots generally concentrated towards the city boundary, while the low-low clusters approached the Taihu Lake. Their locational determinants were identified by spatial regression. In particular, firms near the city boundary and county road were prone to discharge more wastes. Lower waste emissions were more likely to be observed from firms with high proximity to freight transfer stations or the Taihu Lake. Dense populous districts saw more likelihood of solid waste emissions. Firms in the neighborhood of rivers exhibited higher waste water emissions. Besides, the control variables (firm size, ownership, operation time and industrial type) also exerted significant influence. The present methodology can be applicable to other areas, and further inform the industrial pollution control practices. Our study advanced the knowledge of determinants of emissions from pollution-intensive firms in urban areas.

## Introduction

For many years, water pollution has remained as one of the most daunting challenges for China [1,2,3]. Over 45% of the major rivers in China have been heavily polluted in the past 40 years [4]. The latest national water quality survey showed that water from 41.2% of the lakes, 35.8% of the river sections, 76.8% of the groundwater wells, and 19.9% of the major reservoirs did not reach the standard quality criteria [5]. It results in the problem that more than 50% of China’s total population consumes chemically and biologically contaminated water (e.g., ammonia, nitrogen, mercury, volatile phenols and petroleum) [6]. It has been evidenced that industrial emission is the major contributor to China’s water pollution [7]. Many pollution-intensive firms in China do not treat their emissions as effectively as those in the other places of the world [8,9]. The declining water environmental quality has raised the concern that China would not sustain its remarkable growth in the long term if industrial pollution continues to deteriorate [10,11]. While managers and policy makers have urgently taken measures to tackle with this massive problem, for policy or regulation to be practical and effective, the foremost and first question to be answered is: what factors determining industrial pollution?

Recent literature has seen growing efforts to characterize determinants of industrial pollution. Majority of these studies used river water quality parameters (e.g., heavy metals and volatile phenols) as indicators of industrial pollution, and attempted to identify the determinants at local or regional scale using statistical approaches. Kang et al. (2010) [12] employed multiple linear regression to analyze the relationship between land cover type and instream heavy metals. Results showed that industrial and mining areas were significant influential factors. Similarly, Yu et al. (2014) [13] found that higher proportions of urban and agricultural land contributed to more instream heavy metal loadings. Su et al. (2013) [14] pointed that the spatial determinants of instream hazardous chemicals included land cover, population, and gross domestic product. Geng et al. (2014) [15] analyzed the determinants of industrial waste water emissions in China using statistical data, and found that economy was the main determinant. Though these studies advanced our knowledge of the determinants of industrial pollution, the influence of firm characteristics remained poorly understood. These studies therefore failed to answer the question that what kinds of firms should be more intensively regulated. Several cases investigated the firm level determinants using an economic approach. They generally focused on the association between firm characteristics and environmental friendly behaviors. Apergis et al. (2013) [16] quantified the impacts of international financial reporting standards and R&D expenditures on the carbon emissions from firms. Cole et al. (2013) [17] demonstrated that carbon dioxide emissions were closely linked to the operation time. Jiang et al. (2014) [10] analyzed the firm-level determinants of the emission intensity of sulfur dioxide, wastewater, and soot in China. Given the environmental negative externalities [18], the geographic locations, which proxy for potential operating costs [19], should be incorporated into the analysis of firm level determinants. Prior literature has reported that location exerted important impact on firms’ operating decisions [20,21]. For example, pollution-intensive firms usually choose to locate in relatively remote areas with convenient transportation, since geographic distance increases the costs to acquire information and supervise [22]. Unfortunately, due to the absence of high-quality data, rather few studies have systematically examined the locational determinants using a geographical approach.

China carried out the first national pollution source census (NPSC) in 2007. In total, 1.58 million industrial sources were surveyed and a firm-level database was established. It recorded the waste emission information from each pollution-intensive firm (e.g., the quantity of discharged waste water, solid waste, waster gas, and noise) and a rich set of firm characteristics (e.g., location, ownership, and registration time). This unique database therefore provides a reliable foundation to characterize the locational determinations of emissions from pollution-intensive firms. This paper aims to fill the gap in the literature using the data of Huzhou City collected during NPSC, a typical industry intensive city in eastern coastal China. Our specific objectives are to: (1) examine the spatial patterns of waste water and solid waste discharged from pollution-intensive firms in Huzhou City; (2) identify the locational determinants of waste water and solid waste emissions; and (3) provide some implications for industrial pollution control.

## Data and Method

### Study area and dataset

**Fig 1:**
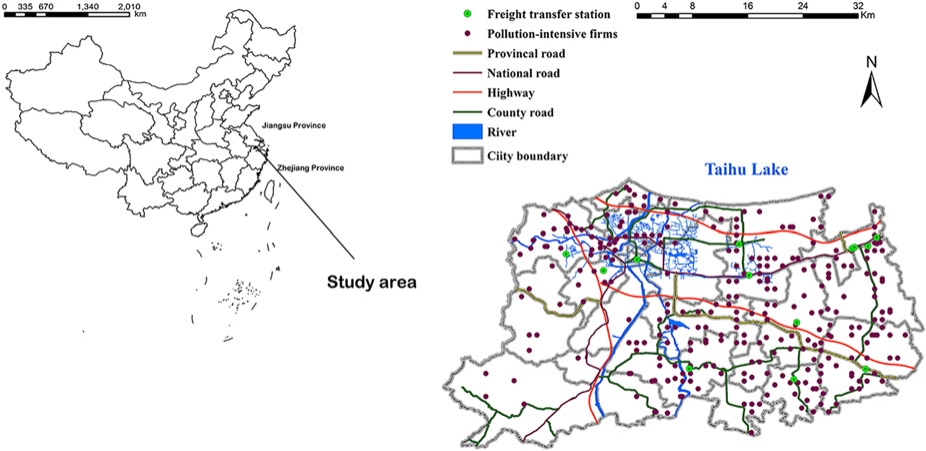
Location of the surveyed firms, river networks, and transportation infrastructure within the Huzhou City, China.

The Huzhou City is located within the Zhejiang Province and borders Jiangsu Province, two of the most urbanized regions in Chinese eastern coast (Fig 1). Given its advantage geographical position and convenient transportation, industrialization has been developing rapidly since the 1980s. Now, it has a complete industrial system, including private, state-owned, and foreign capital enterprises, and has many industrial dense zones. Though the fast industrialization has promoted economic boom in Huzhou City, it becomes the major source of the local environmental pollution. Statistics showed that industrial waste water emissions increased from 70.0 in 1995 to 119.8 million tons in 2012. During the NPSC in 2007, the government investigated the emissions from 717 pollution-intensive firms within the city (Fig 1). According to the NPSC, these firms were selected because they represented the local industrial activities in the broader economy. We were accessed to the census data [23] of waste water and solid waste from these 717 firms. The descriptive statistics were shown in Table 1.

**Table 1:** Descriptive statistics of the dataset (N = 717).

Variables	Maximum	Minimum	Mean	Standard deviation	Definition
Waste water	3504000	2.1	19699	1.2	Amount of discharged waste water (ton)
Solid waste	251.3	2.2	10.2	31.6	Amount of discharged solid waste (ton)
Private	1	0	0.28	0.39	Dummy variable, = 1 if the firm is private owned; = 0, otherwise
State	1	0	0.19	0.35	Dummy variable, = 1 if the firm is state owned; = 0, otherwise
Foreign	1	0	0.15	0.31	Dummy variable, = 1 if the firm is foreign owned; = 0, otherwise
Limited	1	0	0.08	0.23	Dummy variable, = 1 if the firm is non-state owned limited; = 0, otherwise
Collectives	1	0	0.21	0.26	Dummy variable, = 1 if the firm is registered as collectives; = 0, otherwise
Public	1	0	0.22	0.29	Dummy variable, = 1 if the firm is registered as public-listed; = 0, otherwise
T_operation	31	1	9.41	7.32	Duration since the registration year
Size	25.4	0.01	1.45	15.7	The number of employees by the end of 2007 (thousand persons)
Resource	1	0	0.38	0.27	Dummy variable, = 1 if the firm belongs to resource intensive industry; = 0, otherwise
Labor	1	0	0.32	0.25	Dummy variable, = 1 if the firm belongs to labor intensive industry; = 0, otherwise
Capital	1	0	0.21	0.19	Dummy variable, = 1 if the firm belongs to capital intensive industry; = 0, otherwise
Technology	1	0	0.13	0.22	Dummy variable, = 1 if the firm belongs to technology and knowledge intensive industry; = 0, otherwise

### Spatial exploratory analysis

Spatial exploratory analysis was employed to analyze the patterns of waste water and solid waste emissions in space. It can capture the “local clusters” in space both visually and statistically and therefore should contribute to more scientific interpretations [24,25]. Local Moran’s I index (Eq (1)) was used to identify the local spatial clusters or spatial outliers of waste emissions. Spatial clusters consist of two categories: (i) high—high clusters indicate high values of waste emissions surrounded by high values; (ii) low-low clusters indicate clustering of low values of waste emissions. Spatial outlier locates within the mixture of low and high values of waste emissions and includes high-low (a high value point is surrounded by low value points) and low-high (a low value point is surrounded by high value points) outliers.

$$I_{i}=\left[\frac{z_{i}}{\left(\frac{\sum_{i}z_{i}^{2}}{n}\right)}\right]\sum_{j}w_{ij}z_{j}(z_{i}=x_{i}-\overset{-}{x})$$ (1)

where *I*_*i*_ represents the similarity in *x* between observation *i* and observations *j* in the neighborhood of *i* defined by a weight matrix *w*_*ij*_.

Weight matrix in spatial exploratory analysis can be divided into two categories: the contiguity matrix and distance matrix. No widely accepted criterion exists for establishing weight matrix. However, the basic rule for matrix specification is to employ fewer neighbors instead of extra neighbors [26]. Meng et al. (2009) [26] argued that sufficient spatial autocorrelation information was incorporated in the nearest neighbor distance matrix for geospatial analysis. The number of nearest neighbors usually accounted for 25% of the total samples [26,27]. We therefore used the nearest neighbor distance (n = 180 points) as spatial weight matrix.

### Selection of potential determinants

Scholars have reported many different determinants of industrial pollution. However, there still existed no formal conceptual model to identify the corresponding determinants. At the firm level, firm ownership, firm size, industry type, and operation time were the most reported influential factors [10,16,17]. At local and regional level, the corresponding determinants are usually divided into two categories: physical and proximity determinants [12–14]. Our study aimed to identify the locational determinants of emissions from pollution-intensive firms using a spatial approach. Considering the data availability, we chose several potential proximity and physical determinants that related closely to geographical locations. We also chose several firm level determinants as control factors, since pollution emissions had close relationships with firm characteristics. The specific reasons for selecting potential determinants were summarized as follows.

Distance to administrative boundary: Industrial pollution has negative externalities; it emerges when the behavior individual does not take the responsibility for pollution and the subsequent damage on others [18]. Industrial pollution in inner city tends to be transferred to the adjacent rural areas, in order to reduce the high environmental costs. We therefore chose distance to the city boundary (D_city) as a potential determinant. Besides, Huzhou City borders on Taihu Lake with Jiangsu Province, one of the most polluted lakes in China. Pollution intensive firms around the Taihu Lake received strict regulations from the government. Therefore, we further selected distance to Taihu Lake (D_lake) as a potential determinant.

Distance to transportation infrastructure: Firms are always located with high proximity to transportation routes but far away from transport hubs, in order to reduce the costs of waste disposal [1,19,28]. We therefore selected several variables: distance to highway (D_high), distance to national road (D_national), distance to provincial road (D_provincial), distance to county road (D_county), and distance to freight transfer station (D_station).

Topological variables: We did not included topological variables into analysis, because the study area is plain with no significant relief.

Variables of negative externality transfer: Disposal approach and environmental impact varies with waste types. When discharging waste water, the firms have to consider the carrying capacity of rivers. Two rivers flow through Huzhou City, named West Tiaoxi River and East Tiaoxi River. Distance to the West Tiaoxi River (D_wriver) and distance to the East Tiaoxi River (D_eriver) were there chosen. Besides, the population distribution should be seriously taken into consideration when discharging solid waste. The population density (Pop) of the district where one firm located was thus selected as a potential determinant.

Control variables: Environmental behaviors of firms have close relationships with firm characteristics [10]. We therefore selected three control variables: ownership (Own), operation time (T_operation) and firm size (Size). Six mutually exclusive types of ownership existed for these firms: privately owned (private), state owned firms (state), foreign owned (foreign), non-state owned limited (limited), collectives (collectives), and public-listed (public). Operation time denotes the duration since the registration year. Firm size refers to the number of employees by the end of 2007. Besides, a firm’s pollution emission is normally influenced by the nature of the industry to which the firm belongs to. One more control variable (industrial type) was therefore chosen. According to the local classification of industrial type of Huzhou City, four industrial types existed for these firms: resource intensive (resource), labor intensive (labor), capital intensive (capital), and technology and knowledge intensive (technology).

### Spatial regression

The ordinary least square (OLS) (Eq (2)) is the most popular tool to identify determinants of industrial pollution. However, industrial pollution always presents spatial autocorrelation, which violates the assumption of OLS [14]. In order to confirm the existence of spatial autocorrelation, the global Moran’s I index [29] was applied to characterize the spatial dependence of waste emissions. Ranging from -1 to 1, a value of 1 denotes the cluster of high or low values (perfect positive spatial autocorrelation), a value of 0 suggests a random pattern (perfect spatial randomness), and a value of -1 indicates a checker board pattern (perfect negative spatial autocorrelation).

**Fig 2:**
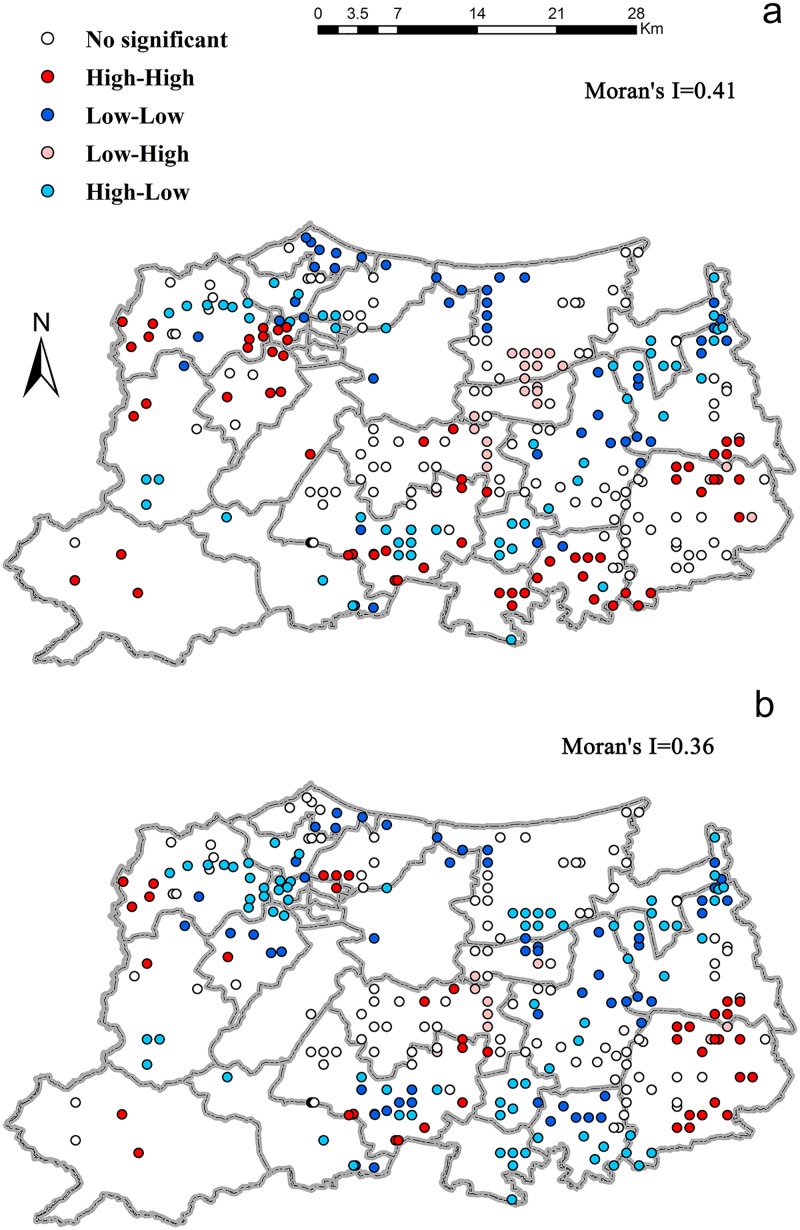
Spatial clusters and outliers, identified by the local Moran’s index, for emissions of waste water (a) and solid waste (b) from pollution-intensive firms in Huzhou City, China.

Considering that waste emissions presented significant spatial autocorrelation (Fig 2), spatial regression was utilized to identify the locational determinants of waste emissions (waste water, and solid waste) from pollution-intensive firms. Spatial regression extends the OLS by incorporating spatial dependence. There typically exist two categories of spatial regression [25]: the spatial lag (Eq (3)) and the spatial error regression (Eq (4)).

Y=λ+X×η+e (2)

$$Y=\lambda +X\times \eta +W_{Y}\times \beta +e$$ (3)

$$Y=\lambda +X\times \eta +e(\text{e}=W_{e}\times \beta +\gamma )$$ (4)

where X is independent variable; Y is dependent variable; η is a vector of coefficients for X; λ and γ are scalar parameters; W_Y_ is the spatial matrix for the dependent variable; W_e_ is the spatial matrix for the error term; β is spatial autoregressive parameter for spatial matrix.

The two regression models include spatial lag and error dependence, respectively [28]. Spatial lag regression suggests a spatial diffusion process, where the influences from the neighborhood should be considered [30]. Spatial error regression denotes the existence of structural factors accounting for the unexplained residuals [27]. Robust Lagrange Multiplier diagnostics [31], which were based on the likelihood ratio test, were used for model specification (spatial lag or spatial error). All the variables were first standardized and normalized before subjected to spatial regression. The quantity of discharged waste water (solid waste) was the dependent variables, and the selected potential determinants were the corresponding exploratory variables. In particular, the traditional variance-in-inflation approach was applied to select the input independent variables, given potential multi-collinearity among the exploratory variables. Spatial regression was performed by GeoDa 0.9.5-i (Beta) [28] using the nearest neighbor distance matrix.

## Results and Discussion

### Spatial patterns of waste emissions

It can be seen from Fig 2 that emissions of waste water and solid waste presented significantly positive autocorrelation in space (Moran’s I >0.35). It implied that the quantity of waste emissions from one pollution-intensive firm was similar to that from its surrounding firms. For waste water (Fig 2a), most high-high clusters concentrated towards the city boundary. Along the river networks, high-low outliers and some high-high clusters could be observed. Low-low clusters could be found in the border of Taihu Lake and Huzhou City. Such results denoted that firms near the Taihu Lake discharged less waste water; however, waste water emissions from those approaching the city boundary were higher. Fig 2b showed the spatial clusters and outliers of solid waste. The high-high clusters generally distributed towards the southeastern and the northwestern boundary. Besides, the high-low outliers were also concentrated near the boundary. It suggested that more solid waste was discharged from the firms with high proximity to the city boundary. Given the distribution of low-low clusters, it can be inferred that firms in the neighborhood of Taihu Lake discharged less solid waste.

### Locational determinants of emissions from pollution-intensive firms

Locational determinants of waste water and solid waste, identified by spatial regression, were listed in Table 2. In particular, 68.1% and 65.7% of the total variances were explained for waste water and solid waste, respectively. Besides, Moran’s I value approached zero for model residuals, signifying that there was no significant autocorrelation in model residuals. All these results denoted that spatial regression was powerful in identifying the locational determinants of emissions from pollution-intensive firms.

**Table 2:** Coefficients of spatial regression for locational determinants of waste emissions from pollution-intensive firms in Huzhou City, China (N = 717).

	Waste water	Solid waste
D_city	-.203**	-.106**
D_lake	.077**	.065**
D_high	ns ^a^	ns
D_national	ns	ns
D_provincial	ns	-.031**
D_county	-.103**	-.209**
D_station	.061**	.204**
D_wriver	-.108**	ns
D_eriver	-.247**	ns
Pop	ns	.137**
private	ns	ns
state	.061**	.041**
foreign	-.224**	-.116**
limited	ns	ns
collectives	-.015**	ns
public	-.044**	-.014**
T_operation	.264**	.146**
Size	.105**	.024**
resource	ns	.134**
labor	.075**	ns
capital	ns	ns
technology	-.115**	-.065**
Model specification	Lag (WY = 2.134**)	Error (Lambda = 3.776)
Constant	2.965	ns
R^2^	.681	.657
Moran’s I for model residuals	.001	.001

**p<0.01

Abbreviations: *distance to the city boundary* (D_city); *distance to Taihu Lake* (D_lake); *distance to highway* (D_high), *distance to national road* (D_national), *distance to provincial road* (D_provincial), *distance to county road* (D_county), and *distance to freight transfer station* (D_station); *distance to the West Tiaoxi River* (D_wriver); *distance to the East Tiaoxi River* (D_eriver); *population density* (Pop); *ownership* (Own); *privately owned* (private); *state owned firms* (state); *foreign owned* (foreign); *non-state owned limited* (limited); *collectives* (collectives); *public-listed* (public); *operation time* (T_operation); *firm size* (Size); *resource intensive* (resource); *labor intensive* (labor); *capital intensive* (capital); *technology and knowledge intensive* (technology); ns: no significant.

Emissions of the two pollutants both had negative correlation with D_city. It denoted that firms near the city boundary were prone to discharge more waste water and solid waste. Such discovery accorded with the previous statement that the negative externalities of environmental pollution were always transferred from urban to rural areas [18,32,33]. The positive coefficient for D_lake should be attributed to the role of environmental regulation. The worsening water quality of Taihu Lake has motivated the managers to place strict regulation on point pollution sources in its neighborhood. Consequently, the firms with high proximity to Taihu Lake were forced to reduce their emissions, through technological innovation, geographical relocation, or even closure. This discovery supported that environmental regulation played an important role in shaping the behaviors of pollution-intensive firms [34,35].

Higher emissions of waste water and solid waste were more likely to be observed in firms near county road, given the negative coefficients for D_county. Besides, D_provincial also had negative relationship with solid waste emissions. Transportation convenience was a critical influential factor for the disposal of wastes in firms [1,19,35]. Firms near transportation routes charged more pollutants because the industrial wastes could be timely transferred to the refuse treatment places. It should be mentioned that the influence of highway and national road was insignificant. It makes sense in that human activities and movement rely heavily on the local transportation networks. Emissions of the two pollutants both had positive correlation with D_station, suggesting that firms with high proximity to freight transfer stations discharged relatively fewer wastes. It should be attributed to it that public transportation hub always requires high standard of environmental quality.

The two variables indicating distance to river both had negative relationship with waste water emissions. Such results demonstrated that firms, discharging high volumes of waste water, generally concentrated towards the rivers. The West Tiaoxi River and East Tiaoxi River were the major local water sources for industrial consumption; however, they became more polluted due to the poor regulation from the government. Firms directly discharged waste water into the two rivers to save treatment costs. Higher probability of solid waste emissions from firms occurred in more populous districts, given the positive coefficient for Pop. These firms were labor-intensive in general; they concentrated in populated districts due to the high demand of labor. Besides, solid wastes are usually transferred to particular sites for processing. Their threat to public health should be lowered after being transferred.

Control variables (ownership, size and operation time) also exerted significant influence. Specifically, state owned firms discharged more waste water and solid waste, while foreign owned firms and public-listed firms discharged fewer wastes. These results were consistent with previous discovery that foreign firms complied with more strict environmental regulation than local firms in China [10]. The state owned firms have more protected policy. For example, they are awarded with more privileges of pollution emissions, since they have more effluent treatment ability and operation capacity [36]. The positive coefficient for T_operation suggested that firms with long operating duration were prone to discharge more waste water and solid waste. These firms were important financial sources for the local government. Consequently, the local government may reduce the environmental regulations on these firms for more fiscal revenue [37]. The positive coefficient for Size suggested that large-sized firms discharged more wastes. Such results were inconsistent the discovery of Jiang et al. (2014) [10] that pollution intensity from small firms were higher than that from large-sized firms. Such inconsistence should be attributed to it that the large-sized firms in the study area were generally long operating state owned firms. Besides, the small-sized firms were strictly regulated by the local government to protect water environment. Table 2 also demonstrated that waste water emissions were higher from labor intensive firms and solid waste emissions were higher from resource intensive firms. On the contrary, waste emissions from technology and knowledge intensive firms were relatively lower. Such results demonstrated that a firm’s pollution emission was normally influenced by the nature of the industry to which the firm belongs to.

### Implications and limitations

The identified locational determinants can be used to predict the potential hotspots of waste emissions, and further inform the industrial pollution control practices. For example, strict regulation should be placed on the firms near the city boundary as well as those with high proximity to county road and the rivers. A report, showing the carrying capacity of the West Tiaoxi River and East Tiaoxi River, should be released to guide the disposal behaviors of the pollution-intensive firms. Besides, as published in some government reports, foreign owned firms were regulated more harshly to reduce industrial emissions in China. Our results demonstrated that such policies could be misguided and should be revised. The present methodology, employing the spatial regression to identify the locational determinants of emissions from pollution-intensive firms, can be applicable to other areas suffering from industrial pollution.

Though this study filled the gap in the literature, it still had several limitations. Firstly, only two typical types of industrial wastes were analyzed, and the locational determinants of the other types (e.g., noise and waste gas) remained unknown. Secondly, there still existed some unexplained variances of the models. It implied that some other determinants were not incorporated into analysis. More detailed study should be further conducted. Thirdly, more advanced regression models can be applied to identify the locational determinants. Further studies can be carried out to compare their predictive abilities and limitations.

## Conclusions

This paper analyzed the spatial patterns and locational determinants of emissions (waste water and solid waste) from pollution-intensive firms in urban areas. Results showed that waste emissions exhibited significantly positive autocorrelation in space. The high-high hotspots of the two pollutants generally concentrated towards the city boundary, while the low-low clusters of the two pollutants approached the Taihu Lake. For waste water emissions, the locational determinants included distance to city boundary, distance to Taihu Lake, distance to county road, distance to freight transfer station, and distance to river. Solid waste emissions were related to six locational variables: distance to city boundary, distance to Taihu Lake, distance to provincial road, distance to county road, distance to freight transfer station, and district population density.

In particular, firms near the city boundary were prone to discharge more waste water and solid waste. Higher emissions of waste water and solid waste were more likely to be observed from firms near the county road. On the contrary, firms with high proximity to freight transfer stations or the Taihu Lake discharged relatively fewer wastes. Besides, higher probability of solid waste emissions from firms occurred in more populous districts. Firms in the neighborhood of rivers discharged more waste water. The control variables (size, ownership, operation time, and industrial type) also exerted significant influences. Specifically, state owned firms discharged more waste water and solid waste, while foreign owned firms and public-listed firms discharged less wastes. In addition, large-sized firms with long operating duration were prone to discharge more waste water and solid waste. The present methodology can be applicable to other areas, and further inform the industrial pollution control practices.

## Supporting Information

S1 Table Geographic coordination of the pollution-intensive firms. (XLSX)

## References

[1] Ma C. Who bears the environmental burden in China—An analysis of the distribution of industrial pollution sources? Ecol. Econ. 2010;69: 1869–1876.

[2] Warren KA, Ortolano L, Rozelle S. Pollution prevention incentives and responses in Chinese firms. Environ. Imp. Ass. Rev. 1999;19: 521–540.

[3] Xue B, Mitchell B, Geng Y, Ren W, Müller K, Ma Z, et al A review on China’s pollutant emissions reduction assessment. Ecol. Indic. 2014;38: 272–278.

[4] Geng Y, Cote R, Fujita T. A quantitative model for optimizing water resources at the industrial park level. Reg. Environ. Change 2007;7: 123–135.

[5] Ministry of Water Resources. 2010 China Water Resources Bulletin. China Water Resources & Hydropower Press, Beijing, 2012.

[6] Ministry of Environmental Protection. 2008 China State of the Environment. China Environmental Science Press, Beijing, 2009.

[7] Kendy E, Zhang Y, Liu C, Wang J, Steenhuis T. Groundwater recharge from irrigated cropland in the North China Plain: case study of Luancheng County, Hebei Province, 1949–2000. Hydro. Process. 2004;18: 2289–2302.

[8] Hu Y, Cheng H. Water pollution during China’s industrial transition. Environ. Dev. 2013;57: 57–73.

[9] World Bank. China 2020: Clear Water, Blue Skies. Washington, DC: The World Bank, 2007.

[10] Jiang L, Lin C, Lin P. The determinants of pollution levels: Firm-level evidence fromChinese manufacturing. J. Compar. Econ. 2014;42: 118–142.

[11] Wei J, Jia R, Marinova D, Zhao D. Modeling pollution control and performance in China's provinces. J. Environ. Manage. 2012;113: 263–270. doi: 10.1016/j.jenvman.2012.08.040 23041518 10.1016/j.jenvman.2012.08.040

[12] Kang J, Lee S, Cho K, Ki S, Cha S, Kim J. Linking land-use type and stream water quality using spatial data of fecal indicator bacteria and heavy metals in the Yeongsan river basin. Water Res. 2010;44: 4143–4157. doi: 10.1016/j.watres.2010.05.009 20599099 10.1016/j.watres.2010.05.009

[13] Yu S, Wu Q, Li Q, Gao J, Lin Q, Ma J, et al Anthropogenic land uses elevate metal levels in stream water in an urbanizing watershed. Sci. The Total Environ. 2014;488–489: 61–69. 10.1016/j.scitotenv.2014.04.061 24815555

[14] Su S, Xiao R, Mi X, Xu X, Zhang Z, Wu J. Spatial determinants of hazardous chemicals in surface water of Qiantang River, China. Ecol. Indic. 2013;24: 375–381.

[15] Geng Y, Wang M, Sarkis J, Xue B, Zhang L, Fujita T, et al Spatial-temporal patterns and driving factors for industrial wastewater emission in China. J. Cleaner Prod. 2014;76: 116–124.

[16] Apergis N, Eleftheriou S, Payne JE. The relationship between international financial reporting standards, carbon emissions, and R&D expenditures: Evidence from European manufacturing firms. Ecol. Econo. 2013;88: 57–66.

[17] Cole MA, Elliott RJR, Okubo T, Zhou Y. The carbon dioxide emissions of firms: A spatial analysis. J. Environ. Econ. Manage. 2013;65: 290–309.

[18] Antoci A, Russu P, Sordi S, Ticci E. Industrialization and environmental externalities in a Solow-type model. J. Econ. Dyn. Contr. 2014;47: 211–224.

[19] Devos E, Rahman S. Location and lease intensity. J. Corpor. Fin. 2014;29: 20–36.

[20] He C. Location of foreign manufacturers in China: Agglomeration economies and country of origin effects. Pap. Reg. Sci. 2003;82: 351–372.

[21] Card D, Hallock K, Moretti E. The geography of giving: the effect of corporate headquarters on local charities. J. Pub. Econ. 2010;94: 222–234.

[22] Duvivier C, Xiong H. Transboundary pollution in China: A study of polluting firms' location Choices in Hebei province. Environ. Dev. Econ. 2011; 18: 1–25.

[23] China Pollution Source Census. http://cpsc.mep.gov.cn/

[24] Chohaney ML. Locational determinants and valuation of Vlach Rom (Gypsy) fortune telling territories in the United States: An integrated application of economic and cultural logics and methods. Appl. Geogr. 2014;53: 32–44.

[25] Su S, Jiang Z, Zhang Q, Zhang Y. Transformation of agricultural landscapesunder rapid urbanization: a threat to sustainability in Hang-Jia-Hu region,China. Appl. Geogr. 2011; 31: 439–449.

[26] Meng Q, Cieszewski CJ, Strub MR, Borders BE. Spatial regression modeling of tree height-diameter relationships. Can. J. For. Res. 2009;39: 2283–2293.

[27] Su S, Xiao R, Xu X, Zhang Z, Mi X, Wu J. Multi-scale spatial determinants of dissolved oxygen and nutrients in Qiantang River, China. Reg. Environ. Change, 2013;13:77–89.

[28] Anselin L. Spatial econometrics: Methods and models. Dordrecht, The Netherlands: Kluwer Academic Publishers, 1988.

[29] Moran P. The interpretation of statistical maps. J. Roy. Stat. Soc. B.1948;10: 243–251.

[30] Ye X, Wu L. Analyzing the dynamics of homicide patterns in Chicago: ESDA and spatial panel approaches. Appl. Geogr. 2011;31: 800–807.

[31] LeSage J, Pace RK. Introduction to Spatial Econometrics. Taylor & Francis/CRC, London, 2009.

[32] Dijkstra BR, de Vries FP. Location choice by households and polluting firms: An evolutionary approach. Eur. Econ. Rev. 2006;50: 425–446.

[33] Petrakis E, Xepapadeas A. Location decisions of a polluting firm and the time consistency of environmental policy. Res. Energ. Econ. 2003;25: 197–214.

[34] Kheder SB, Zugravu N. Environmental regulation and French firms location abroad: An economic geography model in an international comparative study. Ecol. Econ. 2012;77: 48–61.

[35] Zhu S, He C, Liu Y. Going green or going away: Environmental regulation, economic geography and firms’ strategies in China’s pollution-intensive industries. Geoforum 2014;55: 53–65.

[36] Wang H, Nlandu M, Benoit L, Susmita D. Incomplete enforcement of pollution regulation: bargaining power of Chinese factories Policy Research Working Paper Series 2756, The World Bank, 2002.

[37] Taguchi H, Murofushi H. Evidence on the interjurisdictional competition for polluted industries within China. Environ. Dev. Econ. 2010;15: 363–378.

